# Clinical and genetic features of a dominantly-inherited microphthalmia pedigree from China

**Published:** 2009-05-08

**Authors:** Changhong Yu, Zhengmao Hu, Jingzhi Li, Ting Liu, Kun Xia, Lixin Xie

**Affiliations:** 1 College of Medicine, Qingdao University, Qingdao, China; 2 State Key Laboratory Cultivation Base, Shandong Provincial Key Laboratory of Ophthalmology, Shandong Eye Institute, Qingdao, China; 3 The State Key Laboratory of Medical Genetics, Changsha, China

## Abstract

**Purpose:**

To evaluate the clinical, histopathologic, and genetic characteristics of a microphthalmia pedigree.

**Methods:**

A five-generation Chinese family with microphthalmia was recruited. Clinical and histological examinations were performed in the affected patients and their family members. Cyrillic software was used to map the pedigree. Genomic DNA was extracted from peripheral blood, and linkage analysis was performed using short tandem repeat polymorphism markers. Two-point LOD scores were calculated using the MLINK program.

**Results:**

Microphthalmia was inherited in an autosomal dominant manner in this family. All nine affected members had hyperopia (mean: +8.00 diopters) and physiologically reduced axis oculi (mean: 19.29 mm) with a visual acuity of less than 0.5. Refractory angle-closure glaucoma occurred in three of them and atrophia bulbi in two. Histological examination showed diffuse degenerated collagen fibers in the scleral stroma. Two-point LOD score linkage analysis excluded all known genetic loci associated with simple microphthalmia in all patients.

**Conclusions:**

Simple microphthalmia was dominantly inherited in this Chinese pedigree with typical phenotypes, which resulted in severe visual deterioration by middle age. A novel locus is predicted to be responsible for the microphthalmia in this family, which may prove a high genetic heterogeneity in microphthalmia.

## Introduction

Simple microphthalmia (OMIM 309700), which refers to a structurally normal but small eye globe, is an uncommon congenital or developmental ocular anomaly. Absent of systemic disease, it is usually bilateral with a short axis and has hyperopia of +7.00 to +13.00 diopters, a high volume ratio of lens to eye, and a high incidence of angle-closure glaucoma [1,2]. Hyperopia can be treated with correcting lenses, but some children may develop irreversible amblyopia if not diagnosed in time. Untreated angle-closure glaucoma is prone to result in blindness. However, complications are common in microphthalmic eyes after any type of intraocular surgery, especially interventions for glaucoma, which may cause severe loss of vision [3]. Moreover, microphthalmia is a clinically heterogeneous developmental disorder. As major causative genes for simple microphthalmia, *CHX10* (Ceh10 homeodomain gene; OMIM 142993),* PAX6* (Paired box gene 6; OMIM 607108), and *MFRP* (Membrane-type frizzled-related protein; OMIM 606227) probably induce the failure of ocular differentiation [4-6]. In addition to these putative genes, several loci have been identified with microphthalmia, mapping to chromosomes 11p, 14q32, and 2q11-q14 [2,7,8].

It has been reported that 3.2%–11.2% of blind children suffered from microphthalmia [9]. The prevalence rate was 1.18/10,000 births in China [10], which is similar to that in the Caucasian population in England [11]. There was no predilection with regard to race and gender [12]. Most microphthalmos cases were sporadic, and a small quantity of cases were inherited in an autosomal dominant or recessive fashion [13]. In this study, we reported a Chinese family with a dominant form of simple microphthalmia and found that there may be a high genetic heterogeneity in microphthalmia.

## Methods

### Clinical examinations

A five-generation Chinese family diagnosed with microphthalmia was recruited at Shandong Eye Institute (Qingdao, China). The research followed the tenets of the Declaration of Helsinki. All 34 family members underwent general physical and complete ophthalmic examinations including refraction, corneal curvature and axial length by A-B scan ultrasonography, slit-lamp biomicroscopic examination, the measurement of intraocular pressure, gonioscopic examination, and optic-disc evaluation.

### Histological examination

Whole eye globes or full-thickness scleras obtained from the affected members during surgery were subjected to light microscopic evaluation (Eclipse E800; Nikon, Tokyo, Japan). Samples were fixed for 12 h in 4% neutral formaldehyde (pH 7.4). After repeated washing in water, a prolonged dehydration in a graded series of ethanol and an immersion with toluene and paraffin wax were performed. Samples were then immersed in prepolymerized epoxy resin and embedded in paraffin wax. Four-micrometer sections were prepared and stained with hematoxylin and eosin.

### Karyotype analysis

Chromosomes of actively proliferating peripheral blood lympholeukocyte cultures were analyzed. Cells in the exponential phase of growth were treated with 0.06 μg/ml colchicine at 37 °C for 3 h before being collected and incubated in a hypotonic salt solution (0.075 M KCl) at 37 °C for 20 min. After the cells were subsequently fixed in cold methanol/acetic acid (3:1 v/v), 1 ml of dispersed cell suspension was smeared evenly on a cold slide, air dried, stained with Giemsa, and observed using a microscope.

### Linkage analysis

Genomic DNA isolated from 3 ml of peripheral blood using the routine phenolic alcohol-chloroform method [14] was diluted to a concentration of 100 ng/ml. Thirty-four individuals including nine affected family members and 25 unaffected family members were collected and subjected to further analysis. Multiple polymerase chain reaction (PCR) amplifications were performed using AmpliTaq Gold DNA polymerase (PE Applied Biosystems, Foster City, CA) in 15 ml of reaction mixtures with a touchdown procedure. The 23 microsatellite repeat markers for assay were from five autosomes (ABI PRISM Linkage Mapping Panels; PE Applied Biosystems). One of each pair of primers was labeled with phosphoramidite fluorescent tags. Thermal cycling in the Amplifier 2720 (PE Applied Biosystems) was performed. The resulting PCR products were analyzed on an ABI 3100 semiautomated sequencer. GS400 size standards were used as internal standards. Alleles read and scored with Genescan and Genotyper software (PE Applied Biosystems) were confirmed by visual inspection, and two-point LOD scores were calculated using the MLINK sub-program from the LINKAGE package of programs under a model of autosomal dominant inheritance with 95% penetrance and a disease-allele frequency of 0.0001. The recombination frequencies between male and female were assumed equal. Allele frequencies for all markers were calculated from an ethnically matched population.

## Results

### Phenotype

**Figure 1:**
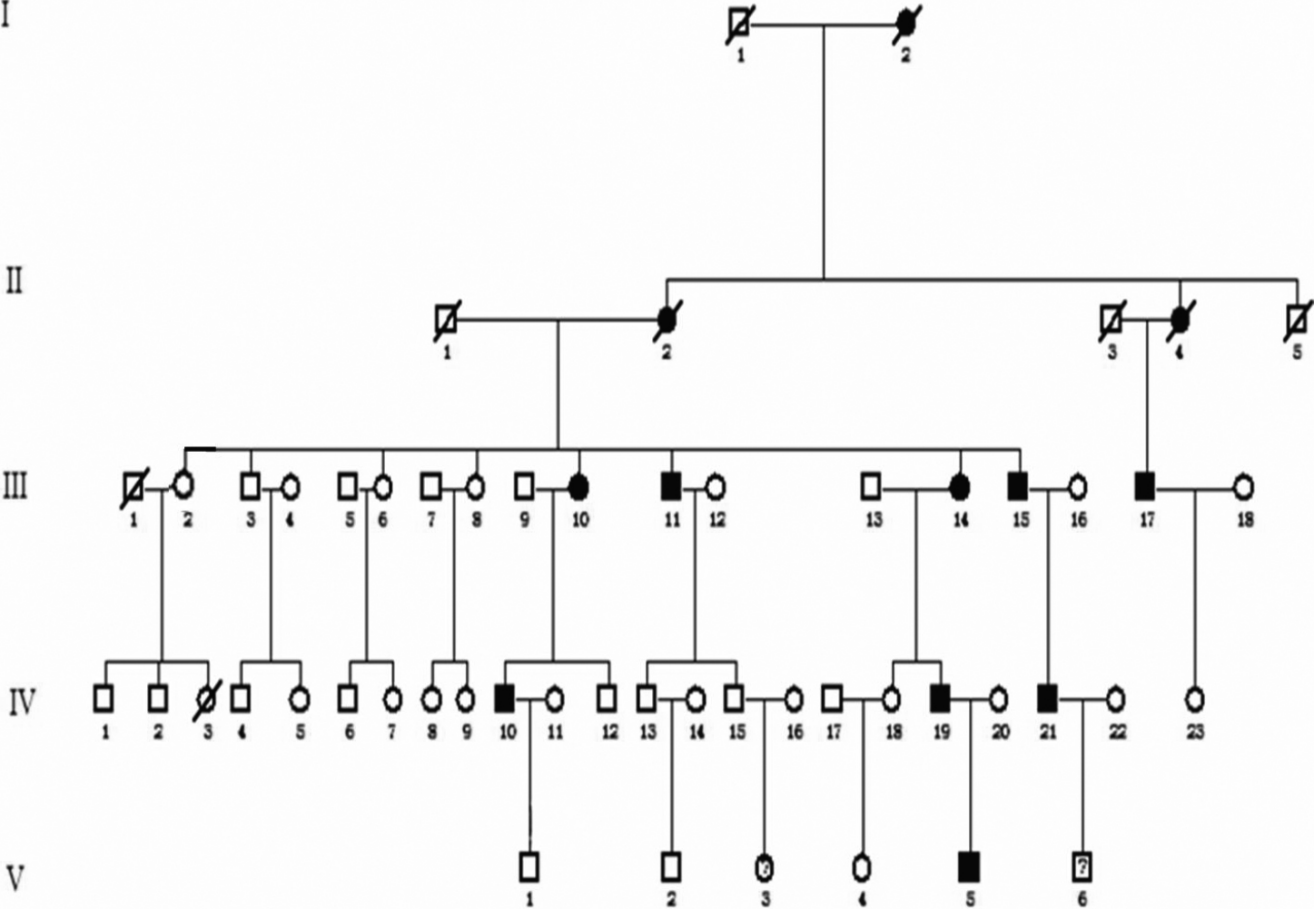
The pedigree of a five-generation Chinese family with autosomal dominant microphthalmia. The symbols in the image are; open circle=female; open square=male; closed circle=affected female; closed square=affected male; open square with backslash=deceased male; closed circle with backslash=deceased female; and open square with question mark; possible affected male.

All family members did not have any other physical anomalies. Nine microphthalmia patients from the ages of 6–66 years (mean: 42.8 years) expressed the same full phenotype as previously reported [2,9]. They were affected by isolated microphthalmia in an autosomal dominant transmission manner in both eyes with onset since birth (Figure 1). Combined hyperopia ranged from +4.50 to +11.50 diopters (mean: +8.00 diopters). The axial length was from 18.22 mm to 20.26 mm (mean: 19.29 mm), which was shorter than the normal length of 23–24 mm. Best corrected visual acuity was less than 0.5 in most eyes. Angle-closure glaucoma or occludable anterior chamber angles were present in three patients who were all older than 50 years of age and was treated by trabeculectomy, but the pathogenetic condition kept developing. In the end, atrophia bulbi occurred in subjects III10 and III15 despite repeated surgeries (Table 1).

**Table 1:** Major clinical data of individuals with microphthalmia in the family.

**Patient no./sex/age (years)**	**Visual acuity (after correction)**	**Diopter/Axis Oculi (mm)**	**Lens A-PD (mm)**	**Increased IOP**	**Glaucoma/patient age when operated**	**Karyotype**
III_10_/F/66	OD: NLP	−	4.96	↑	+/60	46,XX
	OS: FC/30cm	+10.00/18.27	4.94	↑	+/59	
III_11_/M/60	OD: 0.5	+9.00/19.28	4.58	−	−	46,XY
	OS: 0.2	+9.50/19.19	4.56	↑	+/58	
III_14_/F/56	OD: 0.5	+10.50/18.47	5.17	−	−	46,XX
	OS: 0.2	+11.50/18.40	5.13	−	−	
III_15_/M/53	OD: 0.2	−/18.22	4.98	↑	+/52	46,XY
	OS: NLP	−	4.96	↑	+/52	
III_17_/M/50	OD: 0.5	+6.50/19.98	4.88	−	−	46,XY
	OS: 0.3	+6.50/19.91	4.90	−	−	
IV_10_/M/40	OD: 0.5	+8.00/19.78	4.03	−	−	46,XY
	OS: 0.5	+8.50/19.66	4.05	−	−	
IV_19_/M/28	OD: 0.8	+4.50/20.25	4.03	−	−	46,XY
	OS: 0.8	+4.50/20.26	3.99	−	−	
IV_21_/M/27	OD: 0.8	+4.50/20.12	3.98	−	−	46,XY
	OS: 0.8	+4.50/20.16	4.01	−	−	
V_5_/M/6	OD: 0.3	+9.00/19.59	3.54	−	−	46,XY
	OS: 0.3	+8.50/19.63	3.58	−	−	

All affected members had hyperopia and physiologically reduced axis oculi with a visual impairment. FC=finger count; NLP=No light perception; Lens A-PD=Lens antero-posterior diameter; IOP=intraocular pressure.

### Impairment of the global structure

**Figure 2:**
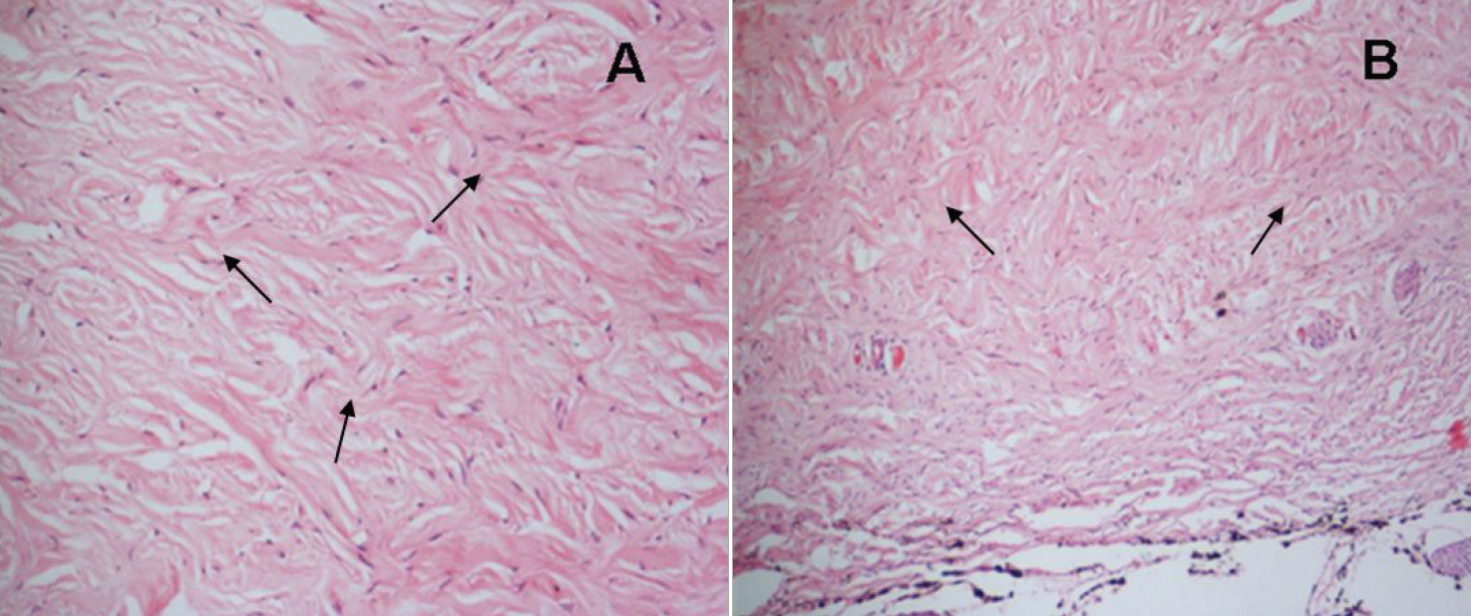
Histological examination of the paraffin sections stained with hematoxylin and eosin. **A**: The diffusely swelled collagen fibers were twisted or frayed (arrows) and mixed with normal fibers in the sclera (Subject III15; magnification, 400X). **B**: The alignment of collagen fibers was disrupted, and there were a lot of coil-like amorphism materials (arrows) in the sclera of an atrophied eye globe (Subject III10; size, 20 mmx18 mmx15 mm; magnification, 100X).

The histological examination showed that the full-thickness scleras were composed of collagen fibers and fibroblasts. The diffusely swelled collagen fibers were twisted or frayed and were mixed with normal fibers (Figure 2A). In the two atrophied globes, which became significantly smaller than the other affected eyes but maintained a relatively normal shape, the alignment of collagen fibers was disrupted and there were a lot of coil-like amorphic materials in the scleras with three times the thickness of a normal sclera. The corneas were thickened as well. The pigment cells proliferated, and bone-like tissues were formed in the eyes (Figure 2B).

### Genetic analysis

The karyotype in the nine affected family members was normal. Two-point LOD scores indicated that all 23 markers were less than 1 under different recombination fractions (Table 2).

**Table 2:** Two-point LOD score linkage analysis with known genetic loci in all patients.

**Marker**	**Position**	**Recombination fraction**						
		**0**	**0.01**	**0.05**	**0.1**	**0.2**	**0.3**	**0.4**
D2S2216	2p11.2-q14.3	−3.30	−0.03	0.55	0.69	0.65	0.45	0.21
D2S160		−18.86	−6.86	−3.77	−2.39	−1.10	−0.48	−0.15
D2S112		−11.99	−6.55	−3.52	−2.17	−0.93	−0.35	−0.08
D2S347		−0.22	−0.22	−0.2	−0.18	−0.14	−0.07	−0.02
D3S1285	3p14.1-p12.3	−5.05	−2.58	−1.64	−1.10	−0.50	−0.19	−0.04
D3S3681		−7.94	−4.46	−2.12	−1.10	−0.25	0.04	0.07
D11S4046	11p15.5-q23.3	−13.67	−3.29	−1.34	−0.63	−0.13	−0.01	−0.03
D11S902		−18.22	−7.62	−4.05	−2.54	−1.17	−0.51	−0.16
D11S904		−9.61	−3.47	−1.36	−0.54	0.07	0.21	0.15
D11S935		−8.95	−2.45	−0.55	0.09	0.44	0.38	0.17
D11S905		−9.28	−1.16	0.04	0.39	0.45	0.25	0.04
D11S4191		−5.47	−1.89	−0.62	−0.19	0.07	0.11	0.07
D11S987		−4.91	−2.06	−0.79	−0.34	−0.05	0.00	0.00
D11S1314		−9.84	−2.60	−0.73	−0.11	0.18	0.08	−0.05
D11S908		−6.10	−3.44	−1.49	−0.76	−0.21	−0.04	0.00
D11S925		−5.57	−1.35	0.03	0.50	0.68	0.50	0.20
D14S258	14q24.2-q32.12	−10.86	−3.68	−1.55	−0.70	−0.05	0.12	0.09
D14S985		−10.10	−3.18	−1.66	−0.99	−0.39	−0.13	−0.01
D14S292		0.54	0.53	0.48	0.41	0.26	0.14	0.05
D14S280		−5.46	−1.87	−0.97	−0.53	−0.15	−0.02	0.00
D15S165	15q13.3-q14	0.12	0.11	0.09	0.07	0.04	0.02	0.00
D15S1007		−11.74	−3.51	−1.39	−0.57	0.02	0.14	0.08
D15S1012		−10.57	−3.25	−1.74	−1.09	−0.51	−0.25	−0.10

All 23 markers were less than 1 under different recombination fractions.

## Discussion

Congenital microphthalmia is rare and results in severe developmental defects of eye globes. Up to 80% of affected individuals are accompanied by physical congenital malformations (multiple malformations) as well as other ocular abnormalities such as coloboma, orbital cyst, and cataract [15]. Isolated microphthalmia is an uncommon condition. Data on these malformations have been scarcely reported, and most cases have been of unknown etiology. In this study, we evaluated an isolated microphthalmia pedigree of Chinese origin. The diagnosis was based upon clinical and imaging criteria and confirmed by histology of the sclera. Establishing a phenotype of this pedigree involved undertaking a comprehensive family and medical history, physical examination, imaging, karyotyping, and molecular genetic testing. The disease pattern was autosomal dominant in this family with nine members affected with microphthalmia. Most patients had serious visual impairment. An unusually high incidence of high hyperopia and frequent occurrence of angle-closure glaucoma were noted. Hyperopia in these affected members was congenital. Complicated glaucoma at middle age represented a late stage of progression of the phenotype. Both were attributed to congenital short ocular axis. These patients experienced a high rate of complications and blindness after intraocular surgery, including two with final atrophia bulbi. Learning disabilities were observed in one-third of the cases. The lens thickening after middle age and the gradually occludable anterior chamber angles could generate refractory glaucoma. In the end, loss of visual acuities would occur in most patients.

The detailed pathogenesis of microphthalmia remains unknown. In the present study, the scleras were subjected for ultrastructural examination. Histologically, collagen fibers in the scleral stroma degenerated, and the sclera became significantly thickened compared to the report of Fukuchi et al. [16]. It is possible that the abnormal collagen fibers directly prevented the early development of eye globes. The atrophied eye globes were obviously small in size, the pigment cells proliferated, bone-like tissue formed, and calcification occurred. These features were consistent with typical microphthalmia.

Moreover, congenital microphthalmia is usually combined with anterior segment dysgenesis [16] and raised intraocular pressure, which may result from eye embryological defects. According to Mann [17], anophthalmia had its genesis early in gestation as a result of the failure to develop the anterior neural tube (secondary anophthalmia) or optic pits to enlarge and form optic vesicles (primary anophthalmia). The genes that regulated eye embryogenesis induction failure could be responsible for microphthalmia. Abnormal migration or proliferation of neural crest cells has been implicated in the development of several anterior segment anomalies and congenital glaucoma syndromes [18,19]. However, the posterior segment of microphthalmic eyes seems to be more frequently affected than the anterior. Weiss et al. [20] reported that postnatal ocular growth was crucial. The decreased size of the optic cup, altered proteoglycans in the vitreous, low intraocular pressure, and abnormal growth factor production may all or in part have a bearing on the pathogenesis of simple microphthalmia while inadequate production of secondary vitreous may result in complex microphthalmia.

Regarding genetic analysis, chromosomal abnormality is usually implicated in microphthalmia with syndromes. The karyotype in this pedigree was normal, so the disease was most probably induced by a mutation of a monogenic mutation. Moreover, genetic defects that underlie the autosomal dominant microphthalmia are still unclear. Microphthalmia has been mapped to different chromosomal regions [21], but this disease appears to be of high genetic heterogeneity, which was proven again in this study. Further investigations on large pedigrees from different genetic backgrounds may help reveal the genetic etiology of microphthalmia [22] and map the phenotype to one member of the genes that direct eye development.

Furthermore, a LOD score of less than 1 with all 23 markers involved did not support any linkage relation in this study, so we excluded the linkage of microphthalmia in this pedigree with the reported candidate genes (*CHX10*, *PAX6*, and *MFRP*) and loci (chromosomes 14q32, 11p, and 2q11-q14). To further identify the gene responsible for this Chinese family, a whole-genome scan analysis is needed. A novel locus is predicted to be responsible for the microphthalmia in this family.
